# Simultaneous bilateral cochlear implants: Developmental advances do not yet achieve normal cortical processing

**DOI:** 10.1002/brb3.638

**Published:** 2017-02-28

**Authors:** Vijayalakshmi Easwar, Hiroshi Yamazaki, Michael Deighton, Blake Papsin, Karen Gordon

**Affiliations:** ^1^ Archie's Cochlear Implant LaboratoryThe Hospital for Sick ChildrenTorontoONCanada; ^2^ Collaborative Program in NeuroscienceThe University of TorontoTorontoONCanada; ^3^ OtolaryngologyThe University of TorontoTorontoONCanada; ^4^ OtolaryngologyThe Hospital for Sick ChildrenTorontoONCanada

**Keywords:** auditory brain, beamformer, bilateral cochlear implant, children, cortical aural preference, cortical lateralization, development, electro‐encephalogram, hearing loss, speech perception

## Abstract

**Background:**

Simultaneous bilateral cochlear implantation promotes symmetric development of bilateral auditory pathways but binaural hearing remains abnormal. To evaluate whether bilateral cortical processing remains impaired in such children, cortical activity to unilateral and bilateral stimuli was assessed in a unique cohort of 16 children who received bilateral cochlear implants (CIs) simultaneously at 1.97 ± 0.86 years of age and had ~4 years of CI experience, providing the first opportunity to assess electrically driven cortical development in the absence of reorganized asymmetries from sequential implantation.

**Methods:**

Cortical activity to unilateral and bilateral stimuli was measured using multichannel electro‐encephalography. Cortical processing in children with bilateral CIs was compared with click‐elicited activity in 13 normal hearing children matched for time‐in‐sound. Source activity was localized using the Time Restricted, Artefact and Coherence source Suppression (TRACS) beamformer method.

**Results:**

Consistent with dominant crossed auditory pathways, normal P1 activity (~100 ms) was weaker to ipsilateral stimuli relative to contralateral and bilateral stimuli and both auditory cortices preferentially responded to the contralateral ear. Right hemisphere dominance was evident overall. Children with bilateral CIs maintained the expected right dominance but differences from normal included: (i) minimal changes between ipsilateral, contralateral and bilateral stimuli, (ii) weaker than normal contralateral stimulus preference, (iii) symmetric activity to bilateral stimuli, and (iv) increased occipital lobe recruitment during bilateral relative to unilateral stimulation. Between‐group contrasts demonstrated lower than normal activity in the inferior parieto‐occipital lobe (suggesting deficits in sensory integration) and greater than normal left frontal lobe activity (suggesting increased attention), even during passive listening.

**Conclusions:**

Together, findings suggest that early simultaneous bilateral cochlear implantation promotes normal‐like auditory symmetry but that abnormalities in cortical processing consequent to deafness and/or electrical stimulation through two independent speech processors persist.

## 1 Introduction

The goal of recommending two cochlear implants (CIs) (Papsin & Gordon, 2008; Peters, Wyss, & Manrique, 2010; Ramsden et al., 2012) in children with bilateral deafness is to promote development of hearing with both ears, and to provide benefits of binaural hearing. This study evaluated whether cortical development with bilateral CIs provided early and simultaneously in children with prelingual deafness parallels development in normal hearing children. Children who receive two CIs simultaneously offer a unique opportunity to evaluate development with electric hearing without the confound of reorganized asymmetries in children receiving their CIs sequentially with long interimplant delays (Gordon, Wong, & Papsin, 2013). Children who receive their second CI after 2 years of listening with one CI show potentially long lasting delayed brainstem responses when stimulated using the second CI relative to the first CI (Gordon, Salloum, Toor, van Hoesel, & Papsin, 2012; Gordon, Valero, & Papsin, 2007; Papsin & Gordon, 2008). Cortically, unilateral hearing leads to abnormal strengthening of pathways from the hearing ear and higher than normal cortical activity in the hemisphere contralateral to the hearing ear (Gordon et al., 2013; Kral, Hubka, Heid, & Tillein, 2013). Such asymmetries manifest in behavioral outcomes such as sound lateralization and speech perception abilities (Gordon, Deighton, Abbasalipour, & Papsin, 2014; Gordon, Henkin, & Kral, 2015; Gordon & Papsin, 2009; Gordon et al., 2013; Jiwani, Papsin, & Gordon, 2016).

Although bilaterally implanted children derive significant benefit when using two CIs compared to one (Gordon & Papsin, 2009), their performance in certain binaural tasks remains poorer and/or abnormal compared to normal hearing children (Litovsky & Gordon, 2016; Steel, Papsin, & Gordon, 2015). The difficulty in integrating input from both ears may relate to factors such as representation of input from individual ears, deafness/hearing loss, hearing experience, and/or lack of integration between bilateral devices. Integration of input from the two ears begins at the superior olivary complex in the lower auditory brainstem (Grothe, Pecka, & McAlpine, 2010). Binaural interactions in the brainstem are present in children with bilateral CIs (Gordon et al., 2012) however, it is not clear how bilateral stimuli are processed cortically. In cats, deafness reduces the number of cortical neurons responding to bilateral stimulation (Tillein et al., 2010), decreases the number of cells showing excitatory responses to both ipsilateral and contralateral stimuli (Tillein, Hubka, & Kral, 2016), and increases the relative proportion of cells responding preferentially to ipsilateral stimuli (Tillein et al., 2016). In addition, cortical preference for contralateral stimuli (Kral et al., 2009, 2013), fine‐structure in propagation of cortical activity (Kral et al., 2009), and sensitivity to binaural cues such as interaural time differences are significantly reduced by deafness (Tillein, Hubka, & Kral, 2011; Tillein et al., 2010, 2016). The reduced spatio‐temporal fine structure in cortical propagation waves results in a more synchronized activation pattern (Kral et al., 2009); this synchrony may be further increased by the rapid onset of electrical pulses provided by the CI (e.g., Hartmann, Topp, & Klinke, 1984). Hearing experience is particularly important for normal development of processing sensory stimuli in the cortex, relative to the brainstem and auditory nerve (Emmorey, Allen, Bruss, Schenker, & Damasio, 2003; Gordon, Wong, et al., 2011; Kral et al., 2013). Sensory deprivation during development could delay appropriate synaptic development and elimination, possibly leading to processing deficits (Kral & Sharma, 2012; Kral, Tillein, Heid, Hartmann, & Klinke, 2005). Thus, physiological evidence suggests that abnormalities in cortical processing of bilateral versus unilateral stimuli in children who are deaf could remain despite simultaneous provision of bilateral CIs. To this end, we compared cortical activity evoked by unilateral and bilateral stimulation in children who received both their CIs in the same surgery, relative to normal hearing children with similar time‐in‐sound experience. Based on physiological evidence and behavioral outcome measures, we hypothesized that changes in cortical activity between unilateral and bilateral stimuli would be smaller in children with bilateral CIs relative to normal hearing children, and that cortical activity for bilaterally presented sounds would be abnormal in children with CI.

## 2 Method

The study protocol (#100000294) was approved by the Hospital for Sick Children's Research Ethics Board, which adheres to the Tri‐counsel Policy on the Ethical Conduct for Research Innovation. Written consent was obtained from parents/guardians.

### 2.1 Participants

**Figure 1:**
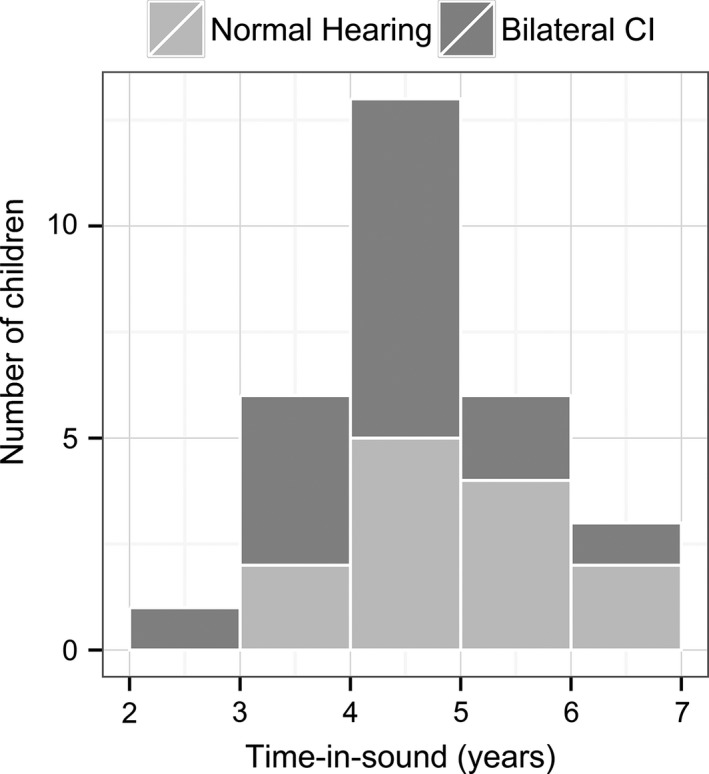
illustrates similar distribution of children's time‐in‐sound (in years) between groups at the time of data collection

Sixteen children (12 boys) with two Nucleus 24 CIs received in the same surgery were recruited. All 16 children had prelingual deafness (additionally progressive in child CI5) and the majority wore hearing aids prior to implantation. Hearing sensitivity prior to cochlear implantation was symmetrical in all children with a mean right‐left pure tone audiometric average (0.5, 1, 2 and 4 kHz) difference of −3.83 dB (SD = 8.87), which is within the ~10 dB test/re‐test error (Stuart, Stenstromb, Tompkins, & Vandenhoff, 1991). Some children had useable residual hearing with hearing aids. Together with preimplant hearing experience and CI experience, the length of time‐in‐sound ranged from 2.04 to 6.03 years. Etiology of deafness, age at implant, age at test, bilateral CI experience, and time‐in‐sound of all 16 children and the group means are provided in Table 1. To compare against cortical activity in a developing auditory system with normal hearing, 13 typically developing children (7 boys) with similar time‐in‐sound experience (equal to their chronological age) and no history of ear, hearing or neurological complaints were recruited. Because children were matched for duration of hearing experience, children in the normal hearing group were ~1 year younger (5.07 ± 0.96 years) than children in the CI group (5.97 ± 0.66 years) (*t*_27_ =  −2.99, *p *= .006). As shown in Figure 1, time‐in‐sound experience between the two groups largely overlapped in range; however, there was a small but significant difference between the 5.07 ± 0.96 years of time‐in‐sound in children with normal hearing and the 4.19 ± 0.91 years in children with CI (*t*_27_ = 2.52, *p *= .018). Older children with CI with longer time‐in‐sound tended to show an emerging N1 response bifurcating the Pci response. These children were not included as the change in waveform is thought to represent myelination of supragranular layers and development of cortico‐cortical connections (Moore & Guan, 2001; Ponton & Eggermont, 2007) which could confound data interpretation. Younger children with normal hearing could not be included due to reduced compliance during testing.

**Table 1:** (A) CI participant demographic data. (B) Group mean demographic data

(A)					
Child	Etiology/risk factor	Age at test (y)	Age at implant (y)	Time‐in‐sound (y)	Bilateral CI experience (y)
CI1	Unknown	5.84	2.70	3.15	3.15
CI2	Unknown	5.54	1.70	4.30	3.85
CI3	Family history	5.29	3.26	2.04	2.04
CI4	Enlarged vestibular aqueduct	6.58	2.85	3.73	3.73
CI5	Incomplete partition type II	5.25	3.16	3.52	2.09
CI6	Unknown	5.88	2.36	3.52	3.52
CI7	Unknown	5.44	1.20	5.04	4.24
CI8	Unknown	6.90	2.83	4.07	4.07
CI9	Premature birth	5.77	1.12	4.65	4.65
CI10	Unknown	5.17	1.04	4.12	4.12
CI11	Congenital Cytomegalovirus infection	5.17	1.06	4.11	4.11
CI12	Low birth weight/NICU stay	6.09	1.09	4.99	4.99
CI13	Unknown	6.72	2.88	4.15	3.84
CI14	Family history	6.85	1.82	5.03	5.03
CI15	GJB2 mutation	7.08	1.05	6.03	6.03
CI16	Unknown	5.98	1.38	4.61	4.61

(B)							
	*N*	Age at test (y)		Age at implant (y)	Time‐in‐sound (y)		Bilateral CI experience (y)
		Mean (SD)	Range	Mean (SD)	Mean (SD)	Range	Mean (SD)
Normal hearing	13	5.07 (0.96)	3.30–6.34	NA	5.07 (0.96)	3.30–6.34	NA
Bilateral CI	16	5.97 (0.66)	5.16–7.07	1.97 (0.86)	4.19 (0.91)	2.04–6.03	4.00 (1.02)

### 2.2 Stimuli

In children with CI, biphasic electrical pulses (pulse width = 25 μs/phase) were delivered from an electrode at the apical end of the implant array at 250 pulses/sec. In children with normal hearing, 100 μs clicks were presented at 250 clicks/sec using Etymotic ER3‐14A insert earphones coupled with a foam tip. Pulse and click trains were 36 ms long and were presented at 1 Hz. Stimuli were presented in the left or right ear in unilateral conditions (hence forth referred to as left‐unilateral and right‐unilateral) and simultaneously to both ears in the bilateral condition. In children with CI, stimulus levels for recording cortical activity were based on stimulus levels necessary to obtain equal amplitude auditory brainstem responses from both CIs when each CI was stimulated individually using single pulses. The levels at which equal amplitude brainstem responses were recorded were further reduced by 10 clinical units (20.96 μA) to account for increases in loudness which could occur through temporal integration between single pulses when presented in pulse trains (36 ms) for cortical response recording (Gordon et al., 2013; Jiwani et al., 2016). These levels were further altered based on perceptually balanced levels when stimuli were presented to both CIs simultaneously (Gordon, Abbasalipour, & Papsin, 2016). In normal hearing children, stimuli were presented at 50 dB above behavioral threshold in each ear that elicited repeatable responses. During testing, the levels for each ear in unilateral conditions matched the levels presented bilaterally.

### 2.3 Response recording

Children wore a 64‐electrode cap and sat in a sound booth. They watched a muted movie of choice with subtitles, played games requiring minimal movement, or read a book during the recording. Electroencephogram (EEG) was recorded using Scan v4.5 with a Synamps‐II amplifier (Compumedics Inc., Charlotte, NC, USA). EEG, referenced to the right earlobe, was sampled at 1000 Hz and bandpass filtered between 0.15 and 100 Hz during recording. Responses were then filtered between 1 and 30 Hz for source localization. The duration of each epoch was 1000 ms and included a prestimulus baseline of 200 ms. Epochs with EEG exceeding ±100 μV at the vertex electrode (Cz) were rejected. At least two replications with a minimum of 100 sweeps/average were obtained per condition.

### 2.4 Localization of cortical activity

**Figure 2:**
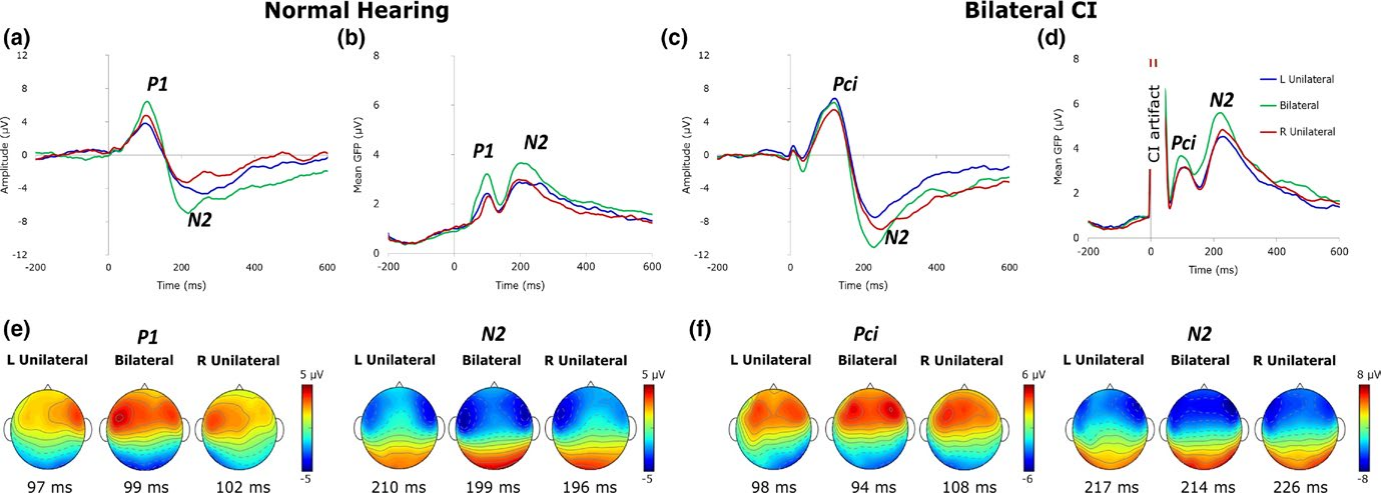
Grand average Cz waveforms and global field power (GFP) in normal hearing children (a, b) and children with bilateral CIs (c, d) to unilateral and bilateral conditions demonstrate immature responses in both groups. (e, f) display average topographic maps at response peaks for P1/Pci and N2 in children with normal hearing and bilateral CIs, respectively. The change between conditions at P1 in children with normal hearing (e) was more distinct than in children with CI (Pci; f). Distribution of EEG activity at N2 was similar in both groups

Cortical activity was localized using the Time Restricted, Artefact and Coherence source Suppression (TRACS) beamformer (details described in Gordon et al., 2013; Jiwani et al., 2016; Wong & Gordon, 2009). In brief, the TRACS beamformer uses an adaptive spatial filter (linearly constrained minimum variance type) to estimate dipole activity from average‐referenced EEG underlying each cortical peak (see P1/Pci and N2 peaks in Figure 2) in ~64000 voxels (3 × 3 × 3 mm/voxel). The time window surrounding each peak for source analysis was chosen individually for each condition and child based on Cz waveforms and global field power (GFP, Figure 2). Dipole activity in all children was constructed using an age‐appropriate head model template from the Montreal Neurologic Institute (MNI) MRI library made using the Template‐O‐matic toolbox (Wilke, Holland, Altaye, & Gaser, 2008). A three‐layer boundary element model mesh was generated based on the head model template to simulate the geometry and conductivity of the brain, skull, and scalp. Source activity in each hemisphere was evaluated by suppressing the other hemisphere (Dalal, Sekihara, & Nagarajan, 2006). In addition, in children with CI, an artifact suppression algorithm was used to suppress the CI‐generated artifact based on activity between −80 to 10 ms in an epoch (Wong & Gordon, 2009). Using this method, 97% of the CI artifact is suppressed while preserving responses beyond the stimulus duration (Wong & Gordon, 2009).

**Figure 3:**
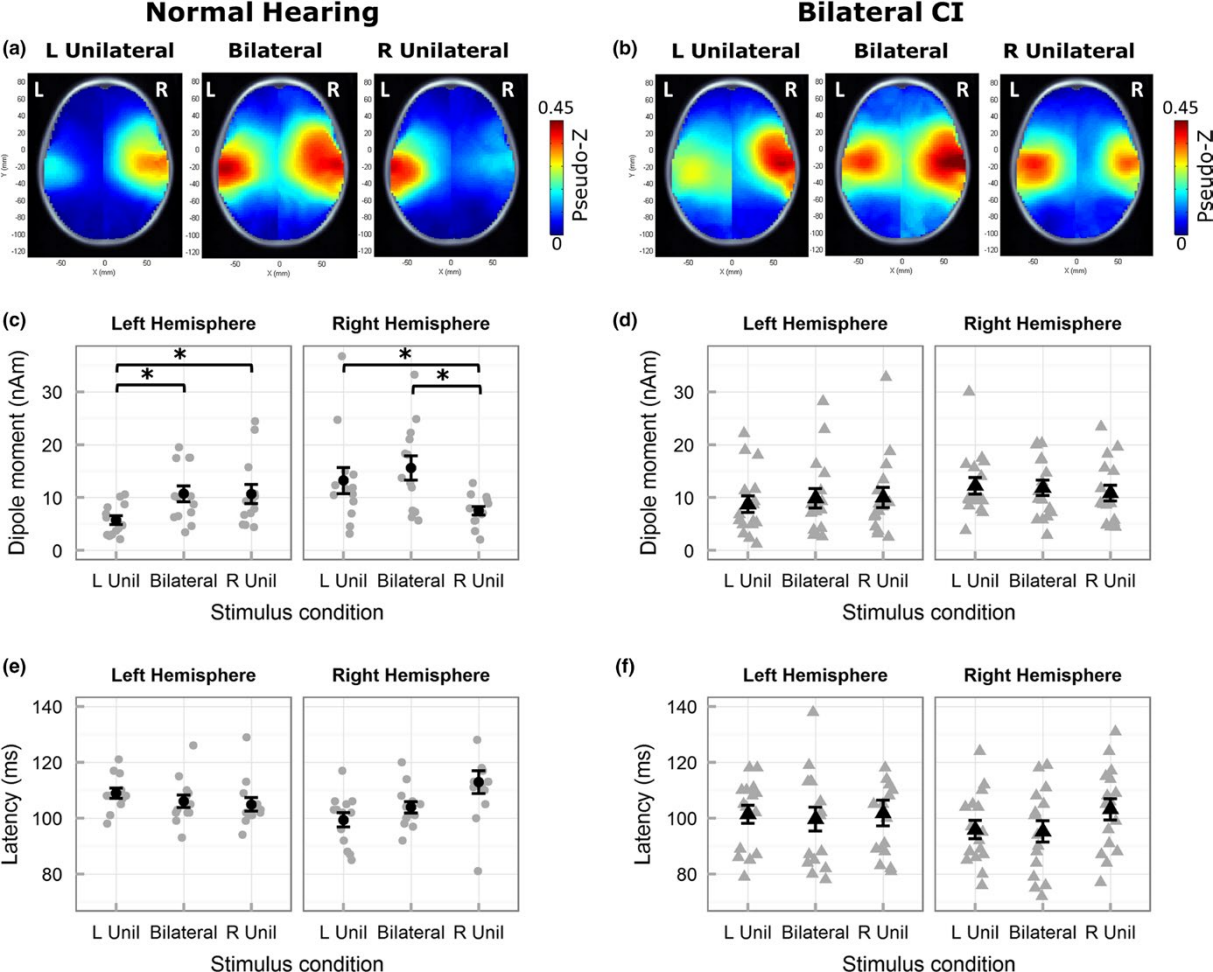
Mean pseudo‐Z maps (only axial images shown) in children with normal hearing (a) demonstrate contralateral cortical activity to unilateral stimulation and bilateral cortical activity to bilateral stimulation during the response peak P1. Mean pseudo‐Z maps in children with bilateral CIs (b) illustrate more bilateral activity during unilateral stimulation. Peak dipole moment (mean in black and individual in gray) to ipsilateral stimulation was lower in left and right hemispheres in children with normal hearing (c), but not in children with bilateral CIs (d). * indicates a significant difference. Peak latencies for ipsilateral stimulation were longer relative to contralateral and bilateral stimulation in both groups (e, f). Error bars represent standard error (SE). Abbreviations L Unil and R Unil represent left‐unilateral and right‐unilateral conditions, respectively

**Figure 4:**
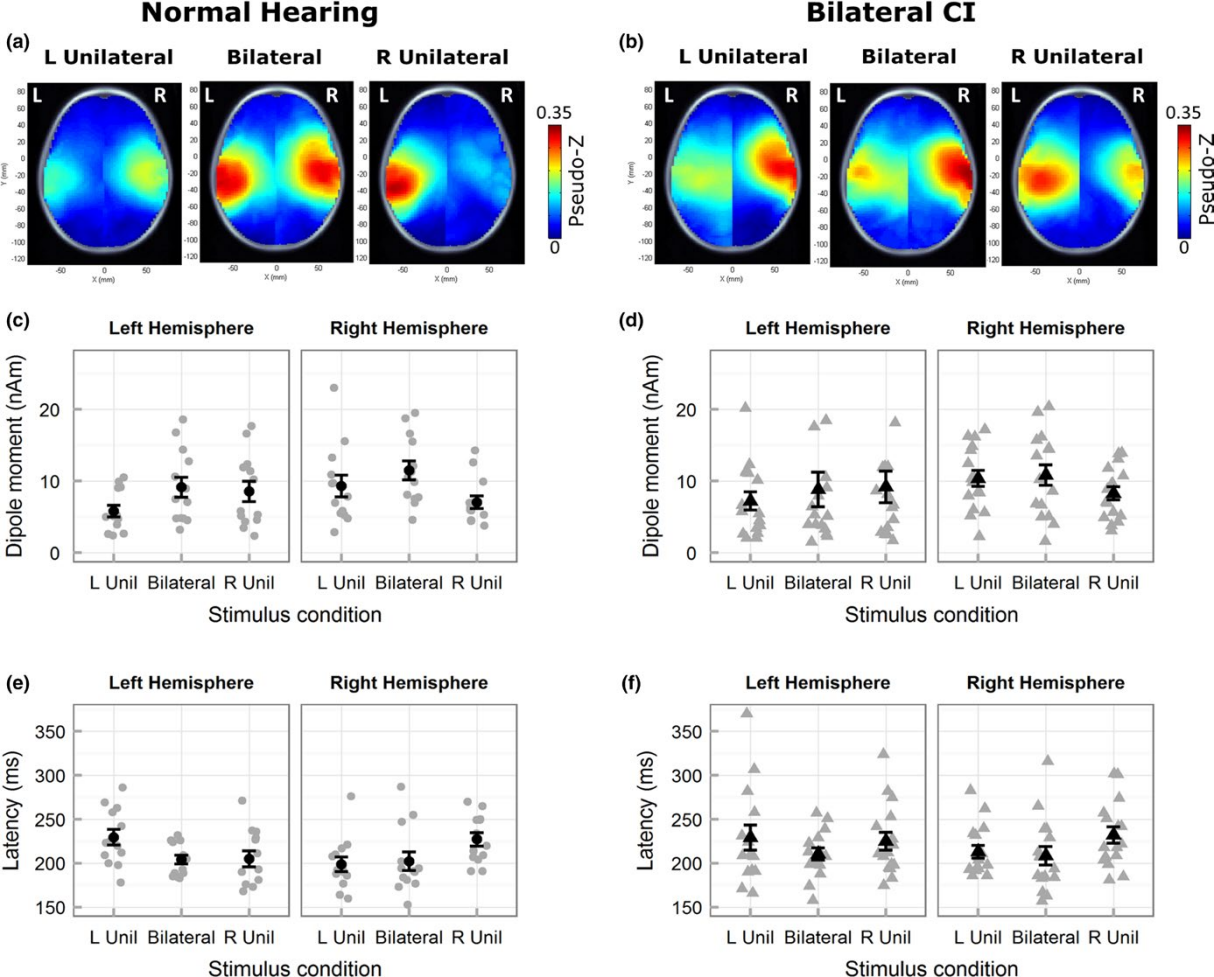
Mean pseudo‐Z maps (axial images) in children with normal hearing (a) and bilateral CIs (b) display similar predominant contralateral activated cortical regions during unilateral stimulation. Peak dipole moment (c, d, mean in black and individual in gray) and peak latency (e, f) illustrate a similar pattern of lower dipole moment and longer latencies for ipsilateral relative to contralateral and bilateral stimulation in both groups. Error bars represent SE. Abbreviations L Unil and R Unil represent left‐unilateral and right‐unilateral conditions, respectively

Activity in each voxel was normalized relative to the prestimulus baseline between −200 and −80 ms using a pseudo‐Z statistic (sample signal mean/standard deviation of prestimulus baseline; Vrba & Robinson, 2001). A threshold pseudo‐Z reflecting baseline brain activity was computed using a one‐tailed omnibus noise *t*‐test (Petersson, Nichols, Poline, & Holmes, 1999) to isolate voxels with above‐baseline activity (Jiwani et al., 2016; Yamazaki et al., unpublished data). Threshold pseudo‐Z was calculated from ± averaging that eliminated time‐locked signals (Gordon et al., 2013). Cortical activity was localized based on pseudo‐Z maps that plot the threshold‐corrected pseudo‐Z for each voxel on MNI head model templates (axial images shown in Figures 3a,b and 4a,b). Hotter colors represent voxels/regions of high signal‐to‐noise ratio and blue represents voxels/regions with below‐baseline brain activity, that is, below the threshold pseudo‐Z.

### 2.5 Peak dipole activity and latency

For each condition and group, average pseudo‐Z maps were generated to identify consistently activated cortical areas (Figure 3a,b and 4a,b). Regions of interest (i.e., auditory cortex) were located using MNI coordinates (X ≤ −55, −35 ≤ Y ≤ 5 & −10 ≤ Z ≤ 20 mm for the left auditory cortex and X ≥ 55, −35 ≤ Y ≤ 5 & −10 ≤ Z ≤ 20 mm for the right auditory cortex; Gordon et al., 2013; Jiwani et al., 2016; Yamazaki et al., unpublished data). The peak dipole was identified as the voxel with the highest pseudo‐Z in each hemisphere's region of interest for a given condition. Further analyses were performed on the dipole moment and associated latency of the peak dipole.

Cortical lateralization was calculated using hemisphere‐specific peak dipole moment using the following formula ‐ ((right‐left hemisphere)/(right + left hemisphere))*100; (Gordon et al., 2013; Jiwani et al., 2016). Positive and negative values indicate right and left lateralized cortical activity, respectively. Sometimes, the peak dipole in one hemisphere was associated with a below‐baseline pseudo‐Z. Its associated peak dipole moment was still included for statistical analysis as this reflects a high degree of lateralization. The associated dipole moments were small (<3 nAm) and therefore representative of low cortical activity in one hemisphere. This was evident in one normal hearing child for P1 (left‐unilateral, left hemisphere), one child with CI for Pci (left‐unilateral, left hemisphere) and three children with CI for N2 (left‐unilateral, left hemisphere (*n *= 2); bilateral, left hemisphere (*n *= 1)). Peak dipoles below baseline pseudo‐Z in both hemispheres were not found in any children. Latencies associated with peak dipole below threshold pseudo‐Z were not included for analyses.

### 2.6 Permutation analyses

Within‐group two‐sided paired permutation tests were used to compare changes in peak dipole activity voxel‐by‐voxel between unilateral conditions, and between unilateral and bilateral conditions using 10000 permutations (Blair & Karniski, 1993; Chau, McIntosh, Robinson, Schulz, & Pantev, 2004). Between‐group two‐sided unpaired permutation tests were used to compare differences between children with normal hearing and bilateral CIs in unilateral and bilateral conditions. To account for multiple comparisons, a Bonferroni correction (*p *=* *.0008; corrected for 62 recording electrodes) was applied (Jiwani et al., 2016).

### 2.7 Speech perception tests

Speech perception was evaluated using tests determined to be age and language appropriate by the child's managing audiologist at the time of testing. For each child, the same test was used in all conditions conducted within a single test session. The following tests were used in the 16 children: Early Speech Perception test (ESP) (*n *= 1), Glendonald Auditory Screening Procedure (GASP) (*n *= 5), Word Identification by Picture Identification (WIPI) (*n *= 2), Multisyllabic Lexical Neighborhood Test (MLNT) (*n *= 4), Phonemic Balanced Kindergarten (PBK) test (*n *= 4). The ESP and WIPI require the child to choose a picture from a group which best represents the target word presented (closed‐set), whereas the child repeats the target word presented in the GASP, MLNT and PBK (open‐set). These tests were carried out in quiet while the child wore each CI at a time. Stimuli were delivered from a speaker at 0‐degree azimuth and percent correct scores were computed. Cortical responses were not evaluated on the same day as the speech perception tests due to the length of test sessions. At the time of speech tests, children had an average of 3.84 ± 1.06 years of bilateral CI experience.

### 2.8 Statistical analyses

Three‐way mixed Analyses of Variance (ANOVA; factors: hemisphere, condition, and group) were conducted for P1/Pci and N2 peak dipole moment and latency. Two‐way mixed ANOVAs (factors: condition and group) were conducted to evaluate differences in cortical lateralization for each peak. Posthoc analyses included two‐tailed paired *t*‐tests corrected for multiple comparison bias using the False Discovery Rate method (FDR; Benjamini & Hochberg, 1995). Corrected *p*‐values are reported and values <.05 were considered significant. Statistical analyses were performed using SPSS 23 (IBM, Armonk, NY, USA).

## 3 Results

### 3.1 Immature cortical responses (P1/Pci‐N2 complex) were recorded in both groups of children

The grand average Cz waveforms and mean GFP along with topographic distributions are shown in Figure 2a–f. Consistent with previous studies (Gordon et al., 2013; Ponton, Eggermont, Kwong, & Don, 2000), children in both groups demonstrated an immature cortical response, with a positive peak (P1 in normal hearing children and Pci in children with CI) circa 100 ms and a following negative peak (N2) circa 210 ms.

### 3.2 P1/Pci: Expected right dominance but increased bilateral activity to unilateral input in CI group

Average pseudo‐Z maps (Figure 3a,b) indicated similar activated regions in both groups. In children with normal hearing, left‐unilateral stimulation elicited right hemispheric dominant activity and right‐unilateral stimulation elicited left hemispheric dominant activity. Bilateral stimulation activated both hemispheres. In children with CI, a similar pattern was observed in the unilateral conditions; however, a hotspot of activity remained clear in the hemisphere ipsilateral to the stimulated side.

Peak dipole moment (Figure 3c,d) followed patterns observed in the pseudo‐Z maps. Generally, higher activity was evident in the right hemisphere in both groups. ANOVA (hemisphere, condition, group) revealed a significant main effect of hemisphere (*F*_1,27_ = 10.69, *p *= .003) with higher average peak dipole moment in the right hemisphere (mean ± SD = 11.84 nAm ± 6.77) than the left (mean ± SD = 9.30 nAm ± 6.35). The main effect of group was nonsignificant (*F*_1,27_ < 0.001, *p *= .996) indicating similar overall peak dipole activity in both groups. The lack of a significant two‐way interaction between hemisphere and group (*F*_1,27_ = 0.39, *p *= .537) indicates that the right hemispheric dominance was similar between the two groups. These data thus suggest normal‐like hemispheric symmetry in children with bilateral CIs with no evidence of abnormal hemispheric dominance shown in children with long periods of hearing with one CI (Gordon et al., 2013).

As plotted in Figure 3c, peak dipole moment was weaker in ipsilateral than contralateral or bilateral stimulation in the children with normal hearing. This difference was not clear in children with CI (Figure 3d). Posthoc analyses following a three‐way significant interaction (*F*_1.52,42.44_ = 4.93, *p *= .018) revealed that, in children with normal hearing, peak activity in each hemisphere was lower for unilateral stimulation of the ipsilateral relative to the contralateral side (right hemisphere: *t*_12_ =  −2.93, *p *= .039; left hemisphere: *t*_12_ =  −3.71, *p *= .012), and bilateral stimulation (right hemisphere: *t*_12_ =  −4.80, *p *< .001, left hemisphere: *t*_12_ =  −4.22, *p *= .006). In contrast, no differences between conditions were observed in each hemisphere of children with bilateral CIs (right hemisphere: right‐unilateral vs. bilateral – *t*_15_ =  −1.09, *p *= .389, right‐unilateral vs. left‐unilateral – *t*_15_ =  −1.23, *p *= .357, bilateral vs. left‐unilateral – *t*_15_ =  −0.35, *p *= .879; left hemisphere: left‐unilateral vs. bilateral – *t*_15_ =  −1.42, *p *= .303, left‐unilateral vs. right‐unilateral – *t*_15_ = 1.47, *p *= .303, bilateral vs. right‐unilateral – *t*_15_ =  −0.13, *p *= .981).

Dipole latencies (Figure 3e,f) showed similar patterns of change between conditions in both groups of children. ANOVA (hemisphere, condition, group) revealed a significant two‐way interaction between condition and hemisphere (*F*_1.88,46.85_ = 12.05, *p *< .001). Like peak dipoles, the main effect of group (*F*_1,25_ = 2.61, *p *= .119) was nonsignificant but unlike peak dipoles, the main effect of hemisphere (*F*_1,25_ = 1.56, *p *= .223) was nonsignificant. Posthoc analyses averaged across the two groups revealed longer ipsilateral latencies in the right hemisphere relative to bilateral (*t*_26_ = 3.38, *p *= .006) and contralateral stimulation (*t*_26_ = 4.03, *p *< .001) and similar latencies for bilateral and contralateral stimulation (*t*_26_ =  −0.88, *p *= .464). No significant differences were found in the left hemisphere (bilateral vs. left‐unilateral – *t*_12_ =  −1.64, *p *= .228, right‐unilateral vs. left‐unilateral – *t*_12_ =  −1.25, *p *= .332, bilateral vs. right‐unilateral – *t*_12_ =  −0.02, *p *= .981).

### 3.3 N2: Expected cortical processing of bilateral and unilateral stimuli in both groups

Average pseudo‐Z maps of N2 (Figure 4a,b) were similar to P1/Pci (Figure 3a,b), however, relatively greater bilateral activity was found in response to unilateral stimulation conditions in both groups. Each hemisphere in both groups showed lower peak activity for ipsilateral stimulation relative to contralateral and bilateral stimulation. ANOVA (hemisphere, condition, group) on peak dipole moment revealed a significant two‐way interaction between condition and hemisphere (*F*_1.99,53.95_ = 8.62, *p *= .001). The main effects of group (*F*_1,27_ = 0.11, *p *= .733) and hemisphere were nonsignificant (*F*_1,27_ = 1.92, *p *= .177) suggesting normal patterns of response at this latency window in children with CIs. Averaged across the two groups, posthoc analyses revealed lower activity in ipsilateral, relative to bilateral (right hemisphere: *t*_12_ =  −4.07, *p *< .001; left hemisphere: *t*_12_ =  −2.69, *p *= .023) and contralateral stimulation (right hemisphere: *t*_12_ =  −3.02, *p *= .015; left hemisphere: *t*_12_ =  −2.59, *p *= .023) in both hemispheres.

Variation in N2 peak latencies (Figure 4e,f), was similar to P1/Pci. ANOVA (hemisphere, condition, group) revealed a significant two‐way interaction between condition and hemisphere (*F*_1.89,45.76_ = 4.47, *p *= .018). Similar to P1/Pci peak latency, the main effects of group (*F*_1,24_ = 1.32, *p *= .261) and hemisphere (*F*_1,24_ = 1.95, *p *= .176) were nonsignificant. Averaged across the two groups, posthoc analyses revealed that, in both hemispheres, peak latencies were significantly longer for ipsilateral relative to bilateral stimulation (right hemisphere: *t*_25_ = 2.51, *p *= .038; left hemisphere: *t*_25_ = 2.67, *p *= .038). Peak latencies did not vary between bilateral and unilateral stimulation of the contralateral side (right hemisphere: *t*_25_ =  −0.30, *p *= .767, left hemisphere: *t*_25_ =  −1.08, *p *= .349). Peak latencies were significantly longer for ipsilateral relative to contralateral stimulation in the right hemisphere (*t*_25_ = 2.82, *p *= .038) but not in the left hemisphere (*t*_25_ = 1.47, *p *= .232).

### 3.4 Limited changes in cortical lateralization across conditions during Pci response window

**Figure 5:**
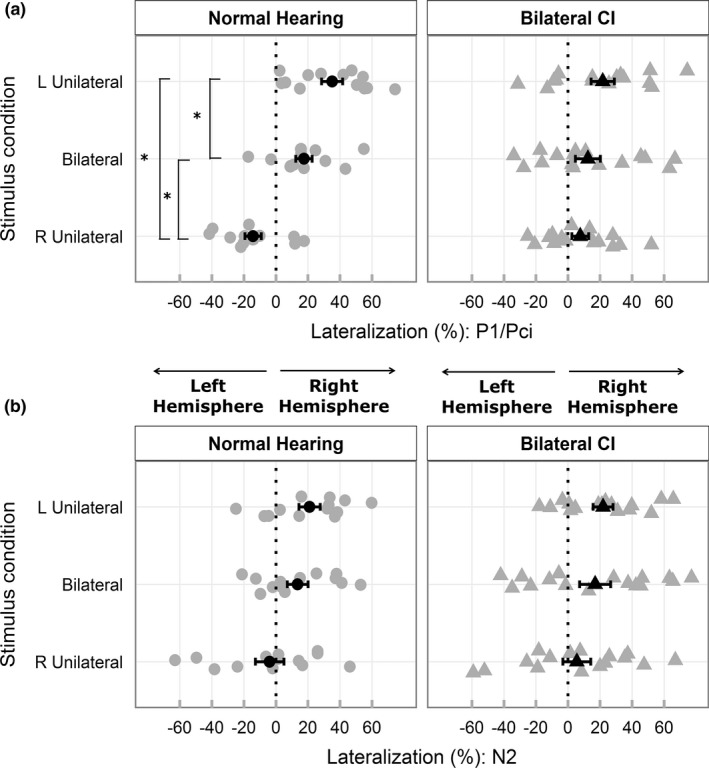
(a, b) display mean (black) and individual (gray) cortical lateralization indices (%) for response peaks P1/Pci and N2, respectively. A significant (indicated by *) progressive increase in cortical lateralization toward the right hemisphere going from right to left unilateral stimulation is evident only in children with normal hearing during P1. Cortical lateralization patterns in N2 resemble that of P1 and are similar between the two groups. Error bars represent SE

Figure 5a and b display cortical lateralization indices for P1/Pci and N2, respectively. Children with CI showed a tendency for right lateralized activity for all conditions on average and limited changes in lateralization between conditions for Pci. A significant two‐way interaction (condition and group; *F*_1.89,51.21_ = 6.41, *p *= .004) was found with no main effect of group (*F*_1,27_ = 0.02, *p *= .868). Posthoc analysis revealed a significant change in cortical lateralization between conditions in children with normal hearing only. Significantly more right lateralized activity was found for left‐unilateral (*t*_12_ = 5.22, *p *< .001) and bilateral (*t*_12_ = 5.12, *p *< .001) relative to right‐unilateral stimulation. Left‐unilateral stimulation led to more right lateralized activity than bilateral stimulation (*t*_12_ = 2.69, *p *= .040). In children with CI, no significant changes between conditions were found: left‐unilateral – bilateral (*t*_15_ = 1.24, *p *= .281); bilateral – right‐unilateral (*t*_15_ = 0.72, *p *= .485); left – right‐unilateral (*t*_15_ = 2.07, *p *= .085).

Changes in cortical lateralization of N2 peak activity were similar between the two groups and partially resembled changes in P1 (Figure 5b). A main effect of condition was found (*F*_1.92,51.92_ = 5.86, *p *= .006) with no group differences (*F*_1,27_ = 0.26, *p *= .614). Averaged across the two groups, significantly more right lateralized activity was found for left‐unilateral relative to right‐unilateral stimulation (*t*_28_ = 3.53, *p *= .003). A tendency for greater right lateralization was evident for bilateral than right‐unilateral stimulation (*t*_28_ = 2.13, *p *= .063), but no differences were found between left‐unilateral and bilateral stimulation (*t*_28_ = 1.05, *p *= .305).

We evaluated hemispheric dominance using one‐sample *t*‐tests (test value of 0; FDR corrected). During P1/Pci, children in both groups showed right hemispheric dominance for left‐unilateral stimulation (normal hearing: *t*_12_ = 5.30, *p *< .001; CI: *t*_15_ = 2.99, *p *= .018). Children with normal hearing showed right hemispheric dominant activity even in bilateral conditions (*t*_12_ = 3.39, *p *= .015), whereas children with bilateral CIs did not (*t*_15_ = 1.59; *p *= .157). Children with CI (*t*_15_ = 1.44, *p *= .170) lacked the left hemispheric dominance for right‐unilateral stimulation evident in normal hearing children (*t*_12_  =  −2.79, *p *= .024). During N2, right dominant lateralization was evident in both groups, but only for the left‐unilateral condition (normal hearing: *t*_12_ = 3.16, *p *= .024; CI: *t*_15_ = 3.51, *p *= .018).

### 3.5 Aural preference for contralateral input is preserved in children with bilateral CIs

**Figure 6:**
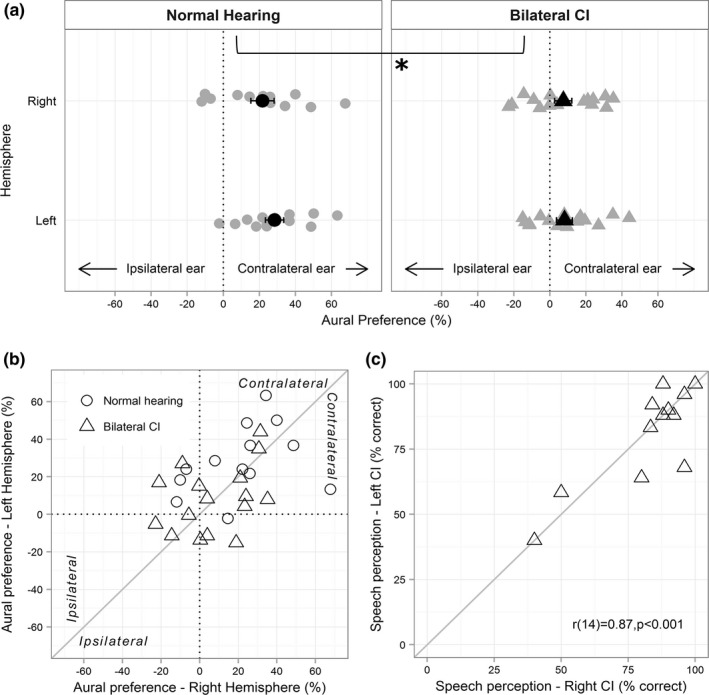
(a) Individual (gray) and group mean (black) aural preference in children with normal hearing and bilateral CIs indicate contralateral aural preference in both groups however significantly lower scores in bilateral CI users (* indicates a significant main effect of group, when averaged across both hemispheres). Error bars represent SE. (b) illustrates the symmetry in aural preference of the left and right hemisphere with greater variability in bilateral CI users. (c) illustrates the positive correlation between the right and left CI speech scores

Unlike cortical lateralization, aural preference (i.e., which ear provides the larger response in the same hemisphere) is not affected by hemispheric dominance. Aural preference in the P1/Pci time window (Figure 6a) was computed using peak dipole moment for contralateral and ipsilateral stimulation within the same hemisphere using the following formula – ((contralateral–ipsilateral)/(contralateral + ipsilateral))*100 (Gordon et al., 2013). Positive values indicate that the hemisphere is more responsive to contralateral than ipsilateral stimulation. As plotted in Figure 6a, many children with bilateral CIs demonstrated expected symmetric and contralateral aural preference. Yet, overall, aural preference in the CI users was less distinct relative to the normal hearing children. A 2‐way ANOVA (hemisphere, group) revealed a significant main effect of group (*F*_1,27_ = 8.23, *p *= .001), but no main (*F*_1,27_ = 0.79, *p *= .382) or interaction effects of hemisphere (*F*_1,27_ = 0.53, *p *= .474). Collapsed across hemispheres, children with normal hearing (mean ± SD = 25.11% ± 4.49) showed significantly greater aural preference relative to children with CI (mean ± SD = 7.78% ± 4.04; *t*_27_ = 2.87, *p *= .008).

A compelling reason for providing two CIs at the same time or with minimal interimplant delay is the protection from abnormal asymmetric reorganization consequent to unilateral hearing. In children with long periods of unilateral hearing through one CI, both auditory cortices prefer the first implanted side (Gordon et al., 2013), that is, one hemisphere develops an abnormal preference for the ipsilateral ear. Data plotted in Figure 6b displays the symmetrical aural preference between the two hemispheres in both groups, consistent with the lack of hemisphere*group interaction in the ANOVA. There is considerable overlap between the two groups and the data cluster around the zero‐difference diagonal albeit with a greater spread in children with CI. The preserved between‐hemispheric symmetry is further supported by the symmetry in functional outcomes, that is, speech perception scores (Figure 6c). Percent correct scores did not vary significantly between the two CIs (mean difference = 1.23%; *t*_15_ = 0.53, *p *= .606) and were highly correlated (*r*_14_  = .87; *p *< .001). Although some children in the CI group showed asymmetrical aural preference patterns, that is, both left and right hemispheres preferring the same ear (left top or right bottom quadrant in Figure 6b), their speech scores were symmetrical, and some aural preference scores overlapped with that of normal hearing children, suggesting normal‐like variations in development. Two children showed >15% difference in speech perception scores between the two CIs. The child (CI13 in Table 1) with the smaller asymmetry in speech scores (16% ‐ closer to the zero‐difference line) falls within the aural preference asymmetry defined by the normal group (right cortex: 0.4%; left cortex: 15.1%), whereas the child (CI5 in Table 1) with the larger difference (28% ‐ > chance performance; Cienkowski, Ross, & Lerman, 2009) showed stronger ipsilateral ear preference for the right hemisphere (right cortex: −21.2%; left cortex:16.9%). Two children also showed slight ipsilateral aural preference for both hemispheres (left bottom quadrant), unlike any of the normal hearing children, but had symmetrical and highly accurate speech perception scores (child 1: right = 83%, left = 83%, child 2: right = 92%, left = 88%), indicating individual variability among children with CI.

### 3.6 Children with bilateral CIs show additional activation in non‐auditory regions

**Figure 7:**
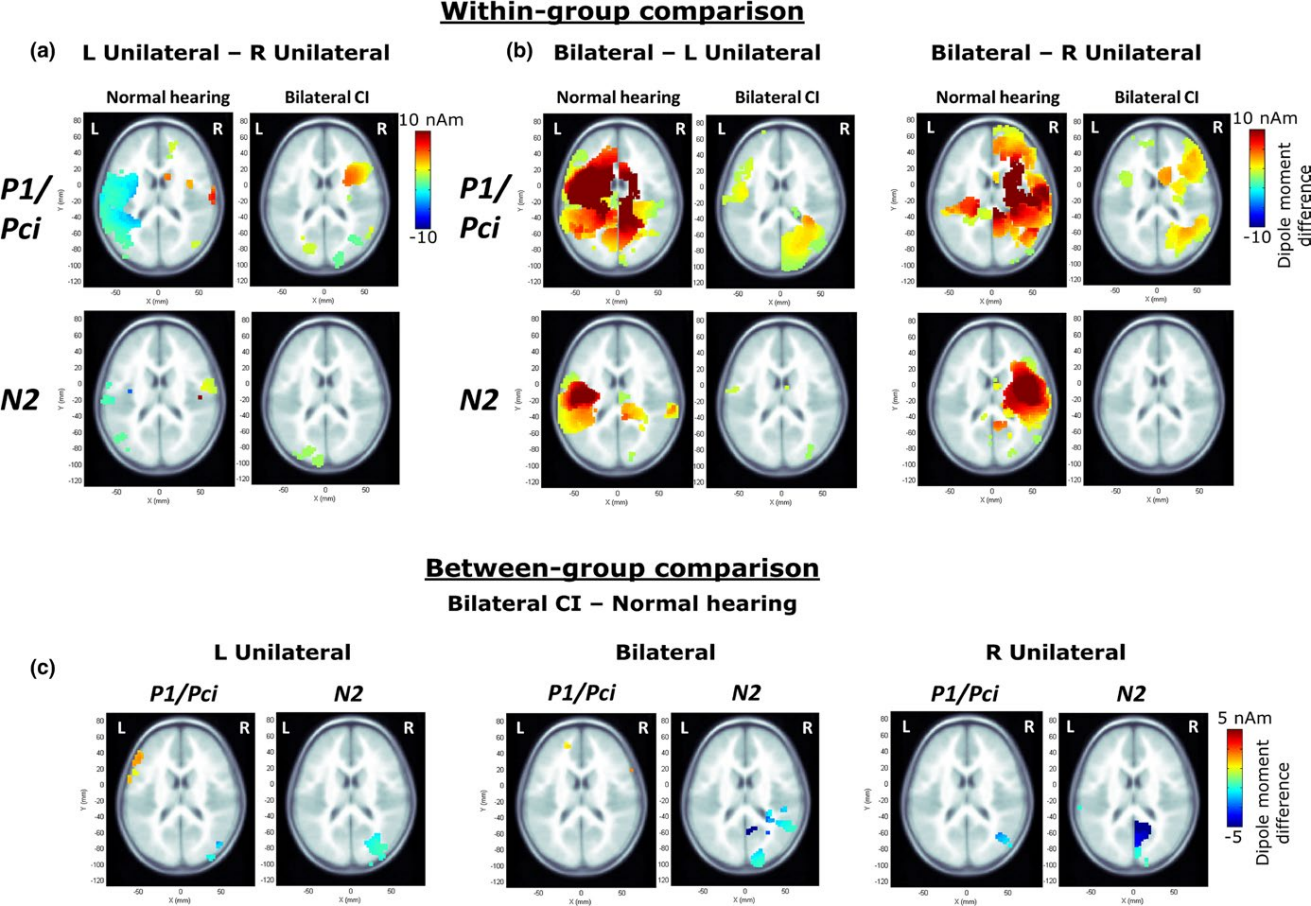
(a and b) plot the voxels showing significant differences in dipole moment between unilateral conditions, and bilateral and unilateral conditions evaluated within each group for each response peak (P1/Pci, N2) using 10000 permutations (corrected for multiple comparisons using a Bonferroni correction; *p *= .05/62 = 0.0008; Jiwani et al., 2016). Only axial images are shown. Hotter colors indicate greater activity in the left‐unilateral condition in (a) and in the bilateral condition in (b). Children with bilateral CIs show fewer differences than children with normal hearing (a, b) and greater activity in the occipital lobe when bilateral conditions are contrasted with unilateral conditions (b). (c) illustrates between‐group differences; hotter colors in the left frontal lobe indicate significantly higher dipole moment in the CI group and cooler colors in the inferior parieto‐occipital regions indicate significantly higher dipole moment in the normal hearing group

Results of permutation analyses are shown in Figure 7. Within‐group analysis compared voxel‐by‐voxel activity between unilateral conditions, and between bilateral and unilateral conditions to evaluate the spread of differences between conditions. Between‐group analyses identified unique areas of cortical activity in bilateral CI users. In children with normal hearing, decreased activity in the ipsilateral temporal cortex to unilateral stimulation during P1 (Figure 7a), shown by decreased dipoles (Figure 3) was confirmed. Comparisons between the two unilateral responses (Figure 7a) revealed decreased activity to left stimulation (blue hotspot) in the left temporal cortex and a small area of reduced activity to right stimulation (red hotspot) in the right temporal cortex. Larger spread of differences in the left hemisphere is consistent with the right hemispheric dominance shown by cortical lateralization measures (Figure 5a; i.e., more contralateral lateralization for left‐unilateral than right‐unilateral stimuli). Symmetric left‐right stimulation differences are seen during the N2 latency window (Figure 5b), but in more focused regions. Similarly, comparisons between bilateral and unilateral stimulation (Figures 3c and 4c) show decreased responses of the latter in the ipsilateral hemisphere (red hotspots) in the normal hearing group with changes at more discrete temporal lobe areas underlying activity in the N2 time window than the P1 window. Consistent with analyses of peak dipole moment and cortical lateralization measures, these differences were nonsignificant in children with bilateral CIs. Children with bilateral CIs showed additional differences in the occipital lobe. Specifically, they had greater activity during bilateral stimulation in the right inferior occipital regions, during Pci (Figure 7b).

Between‐group comparisons, plotted in Figure 7c, indicate largely similar activation patterns in children using bilateral CIs relative to normal with small areas of differences in non‐auditory regions. Children with bilateral CIs had increased activity in a focused region of the left frontal lobe to left‐unilateral and bilateral input. Small decreases were found in children with CI in the right inferior and middle occipital gyrus during left‐unilateral stimulation, and in the fusiform gyrus of the right temporal lobe, middle occipital gyrus and left inferior parietal lobe during right‐unilateral stimulation. During the N2 latency window, lower than normal activity was evident in cuneus and precuneus regions in children with bilateral CIs. The smaller range and regions of differences in the between‐group comparisons compared to within‐subject comparisons may be attributed to individual variability.

## 4 Discussion

This study assessed whether bilateral simultaneous cochlear implantation promotes normal‐like development by measuring changes in cortical activity to unilateral and bilateral stimulation. The cohort of children provided with two CIs without delay provides the first opportunity to evaluate electrically driven auditory plasticity in the absence of reorganized asymmetries due to prior unilateral CI use. Present findings support development of normal‐like symmetry in right and left cortical activity in children with two CIs received simultaneously. However, children with bilateral CIs demonstrated lower cortical preference for contralateral stimulation and smaller within‐ and between‐hemispheric differences across conditions relative to normal. In addition, children with bilateral CIs showed deviant activation in non‐auditory areas.

### 4.1 Bilateral simultaneous cochlear implantation promotes normal‐like symmetrical development and conduction of auditory stimuli

The similarity to normal in right dominance during the Pci time window and symmetrical activity during the N2 window in children with bilateral CIs reinforces recommendations of providing bilateral CIs with short interimplant delays (Gordon & Papsin, 2009; Papsin & Gordon, 2008; Ramsden et al., 2012). Abnormal asymmetric strengthening of pathways from one side (Gordon et al., 2013; Kral et al., 2013) and consequent asymmetric aural preference for that side (Gordon et al., 2013, 2015) did not occur in most children who received their bilateral CIs simultaneously (Figures 3d, 4d and 6). Similar development of bilateral auditory pathways is further supported by symmetric speech perception abilities (Figure 6c). This is unlike asymmetries demonstrated by sequentially implanted children reported previously (Fitzgerald, Green, Fang, & Waltzman, 2013; Gordon & Papsin, 2009; Illg et al., 2013; Jiwani et al., 2016; Peters, Litovsky, Parkinson, & Lake, 2007; Scherf et al., 2009; Sparreboom, Snik, & Mylanus, 2011; Zeitler et al., 2008).

Peaks of cortical activity in children with bilateral CIs occurred at normal latencies across conditions (Figures 3e,f and 4e,f). This suggests that early sensory restoration through two CIs promotes axonal (myelination) and synaptic development up to and within the auditory cortical network, which is otherwise affected by deprivation (Emmorey et al., 2003; Gilley, Sharma, & Dorman, 2008). Earlier latencies for contralateral than ipsilateral stimulation during P1/Pci and N2, is consistent with previous studies (Kral et al., 2013; Ross, 2005; Tiihonen, Hari, Kaukoranta, & Kajola, 1989) and may be explained by the greater number and higher transmission efficiency of crossed than uncrossed fibers (Jancke, Wüstenberg, Schulze, & Heinze, 2002; Malmierca & Hackett, 2010). The similarity between peak latencies for bilateral and contralateral stimuli (Tiihonen et al., 1989), and the asymmetry between hemispheres for contralateral‐ipsilateral stimulus differences is also consistent with previous studies (Joutsiniemi, 1988; Ross, 2005). The contralateral‐ipsilateral latency difference was significant in the right hemisphere with a similar trend in the left, perhaps reflecting effects of stimulation side. This is supported by a tendency for longer peak latencies to right stimulation than left averaged across hemispheres and groups (P1/Pci: 105.36, 100.83 ms; N2: 222.93, 217.63 ms for right and left stimulation, respectively).

### 4.2 Right hemispheric dominance is evident for processing non‐speech stimuli in unilateral and bilateral conditions

A main effect of hemisphere for P1/Pci, with higher peak dipole moment in the right hemisphere compared to the left was found in both groups (Figure 3c,d). In children with normal hearing, this was also reflected in asymmetric lateralization indices during left and right unilateral stimulation, and significant right lateralization (>0) during bilateral stimulation (Figure 5a). In children with CI, a rightward lateralization was evident in all conditions, on average (Figure 5a).

The right hemispheric dominance in unilateral and bilateral conditions may reflect functional hemispheric asymmetries (Hine & Debener, 2007), and possibly arise from imbalanced representations of ipsilateral and contralateral hemifields in each hemisphere (Salminen, Tiitinen, Miettinen, Alku, & May, 2010). Right hemispheric dominance has been reported in adults through greater contralateral lateralization for left ear stimuli than right (Hine & Debener, 2007; Jin, Ozaki, Suzuki, Baba, & Hashimoto, 2008; Ross, 2005; Scheffler, Bilecen, Schmid, Tschopp, & Seelig, 1998; Stecker, McLaughlin, & Higgins, 2015) and during bilateral stimulation (Johnson & Hautus, 2010; Ross, 2005). While lateralized activity for unilateral stimulation occurs due to the predominance of crossed afferent auditory fibers (Gordon et al., 2013; Hine & Debener, 2007; Khosla et al., 2003; Pantev, Ross, Berg, Elbert, & Rockstroh, 1998; Ross, 2005; Scheffler et al., 1998; Stecker et al., 2015; Tiihonen et al., 1989), right lateralized activity during bilaterally matched stimuli may passively reflect right hemispheric dominance in spatial processing (Johnson & Hautus, 2010; Kaiser & Lutzenberger, 2001; Magezi & Krumbholz, 2010; Salminen et al., 2010; Spierer, Bellmann‐Thiran, Maeder, Murray, & Clarke, 2009).

The tendency for right‐lateralized activity (for all conditions including right unilateral stimulation; Figure 5a) in children receiving bilateral CIs simultaneously also resembles emerging right hemispheric specialization for low‐frequency tones in normal hearing children (Yamazaki et al., unpublished data). This may either be due to the apical site of stimulation in children with CI or stimuli inducing a tonal percept (Zatorre & Belin, 2001), as opposed to spectrally broadband clicks which might sound more noise‐like to children with normal hearing. Thus, tones might have evoked more similar results between the two groups than did the clicks used here.

Right hemispheric dominance and group differences were not evident during the N2 latency window. Variations between the response peaks may reflect independent processing stages (Rugg & Coles, 1995), sensitivity to different stimulus features (Ceponiené, Alku, Westerfield, Torki, & Townsend, 2005; Ceponiené, Torki, Alku, Koyama, & Townsend, 2008; Key, Dove, & Maguire, 2005), developmental trajectories (review by Ponton & Eggermont, 2007; Ponton et al., 2000; Ponton, Eggermont, Khosla, Kwong, & Don, 2002), and/or contributing sources (O'Donnell et al., 1993; Ponton et al., 2002). Peak latencies suggest that both P1/Pci and N2 reflect reverberant cortical activity (cortico‐cortical and/or thalamo‐cortical loops), since the first volley into the auditory cortex occurs at ~20 ms (Lee, Lueders, Dinner, Lesser, & Hahn, 1984). However, interhemispheric differences in unilateral and bilateral stimuli appear less pronounced during N2 (greater ipsilateral spread in 5B vs. 5A) in both groups perhaps reflecting a later and more endogenous stage in sound processing (Rugg & Coles, 1995). Development of activity underlying the generators of N2 is not understood in CI users. Hemispheric differences during N2, as shown in this study, appear to be typically developed in children who received their two CIs simultaneously for unilateral and bilateral stimuli.

Although hemispheric dominance was similar between the two groups during the P1/Pci time window, the degree and direction of lateralization during this peak showed clear demarcation of stimulus conditions only in normal hearing children (Figure 5a). Limited changes between unilateral conditions, and unilateral and bilateral conditions in children with bilateral CIs may have implications for differentiating sounds between‐ and within‐hemifields consisting of level and timing differences. This could therefore lead to deficits in spatial hearing, as reported in perceptual experiments (Gordon et al., 2014; Zheng, Godar, & Litovsky, 2015).

### 4.3 Lower contralateral aural preference in children with bilateral CIs could reflect effects of deafness and/or CI use

Cortical activity patterns seen in children with CI during Pci resemble that of congenitally deaf cats. Lower than normal preference for contralateral input is evident in naïve auditory cortices of congenitally deaf cats (Kral et al., 2009, 2013; Tillein et al., 2010, 2016), suggesting that contralateral dominance is activity dependent. Lower contralaterality is attributed to higher than normal ipsilateral activity (Kral et al., 2009; Tillein et al., 2016), possibly arising from a greater proportion of cells showing ipsilaterally driven excitatory responses (Tillein et al., 2016). Another possible contributor is deafness‐related reduced or immature inhibition driving greater excitability (Kotak, 2005; Kotak, Takesian, & Sanes, 2008). Although quite variable, children with bilateral CIs had higher than normal average ipsilateral peak activity in both hemispheres (Figure 3c vs. d). High variability in children with CI observed previously has been attributed in part to the varied etiology of deafness (Gordon, Tanaka, et al., 2011; Gordon, Wong, et al., 2011). Although children with CI in this study had ~4 years of time‐in‐sound, the resemblance of cortical activity in naïve cortices of deaf cats suggests that auditory deprivation during early years of cortical development (Huttenlocher & Dabholkar, 1997) may still impact development of cortical processing with restored hearing. Reduced aural preference could also reflect the nature of electric stimulation that elicits more synchronized activity across broader neural populations compared to acoustic stimulation (Hartmann et al., 1984; Kral et al., 2009). Therefore, a combination of the effects of deafness and electric stimulation may contribute to lower contralateral aural preference in children with CI.

### 4.4 Maladaptive cortical processing of auditory stimuli is evident in children with CI

Bilateral relative to unilateral stimulation induced greater activity in right inferior occipital regions in children with CI especially during Pci compared to normal hearing, suggesting abnormal recruitment of visual cortical areas while processing bilateral stimuli (Figure 7b). This may indicate greater reliance on vision in children with CI when hearing sounds bilaterally possibly to calibrate their auditory space, because: (i) spatial processing with vision may increase accuracy during conflicting auditory cues (review by King, 2009), (ii) vision loss could lead to auditory spatial processing deficits (Zwiers, Van Opstal, & Cruysberg, 2001), and (iii) congenitally blind adults show recruitment of right hemisphere extrastriate occipital areas during sound localization (Weeks et al., 2000). Children with CI also rely more on visual cues for speech understanding (Schorr, Fox, van Wassenhove, & Knudsen, 2005), and CI users show enhanced audio‐visual integration positively impacting post‐CI outcomes (Giraud, Price, Graham, & Truy, 2001; Strelnikov et al., 2013). It is therefore possible that children with bilateral CIs rely on extra‐auditory facilitation for processing bilaterally presented sounds resembling their everyday listening situations (Easwar, Sanfillipo, Papsin, & Gordon, 2016).

Between‐group comparisons revealed lower cortical activity in the right parieto‐occipital regions in children with bilateral CIs (Figure 7c). Reduced activity in all three stimulus conditions may reflect task‐related distinctions and/or abnormal sound processing. Children with hearing loss have higher tendencies for visual attention deficits despite restored audibility with CIs (Quittner et al., 2009; Yucel & Derim, 2008). Thus, it is possible that visual distractions during recording (e.g., video watching), although unrelated and unsynchronized with auditory stimuli, elicited different parieto‐occipital activity in the two groups. The “division of labor” hypothesis suggests that children with CI need to visually scan the environment more often than normal, leaving insufficient resources for normal visual processing (Quittner et al., 2009; Smith, Quittner, & Osberger, 1998). Alternatively, lower visual activity reflects abnormalities in sound processing even during passive listening in children with CIs. Altered activity in the precuneus, an area involved in sensory integration (Cavanna & Trimble, 2006), has been shown in adolescents with CI (Jiwani et al., 2016). Lower activity in the inferior parietal lobe may indicate deficits in spatial memory of auditory sounds and monitoring of auditory space (Alain, He, & Grady, 2008). Higher left frontal lobe activity in children with CI, also shown previously (Jiwani et al., 2016), implicate additional attention (e.g., Kane & Engle, 2002) for sound processing. In summary, these differences illustrate maladaptive development of cortical sound processing possibly due to deafness and/or compensatory mechanisms developed over time to improve listening with limited acoustic cues provided by CIs. In contrast with P1/Pci peak dipole moment (Figure 3b,c), between‐group differences were not found in auditory regions (Figure 7c). This reflects both the smaller abnormalities found in auditory areas of children using bilateral CIs and the increased variability in these areas in this group.

## 5 Conclusion

Results from this study demonstrate that: (i) early bilateral simultaneous implantation promotes normal‐like symmetry in auditory pathways and expected right dominance in auditory processing, but (ii) with persistence of abnormal and maladaptive cortical processing of sounds possibly due to the effects of deafness and/or shortfalls of present auditory prostheses.

## Conflicts of Interest

None to declare.

## References

[brb3638-bib-0001] Alain, C. , He, Y. , & Grady, C. (2008). The contribution of the inferior parietal lobe to auditory spatial working memory. Journal of Cognitive Neuroscience, 20, 285–295. 18275335 10.1162/jocn.2008.20014

[brb3638-bib-0002] Benjamini, Y. , & Hochberg, Y. (1995). Controlling the false discovery rate: A practical and powerful approach to multiple testing. Journal of the Royal Statistical Society Series B (Methodological), 57, 289–300.

[brb3638-bib-0003] Blair, R. C. , & Karniski, W. (1993). An alternative method for significance testing of waveform difference potentials. Psychophysiology, 30, 518–524. 8416078 10.1111/j.1469-8986.1993.tb02075.x

[brb3638-bib-0004] Cavanna, A. E. , & Trimble, M. (2006). The precuneus: A review of its functional anatomy and behavioural correlates. Brain, 129, 564–583. 16399806 10.1093/brain/awl004

[brb3638-bib-0005] Ceponiené, R. , Alku, P. , Westerfield, M. , Torki, M. , & Townsend, J. (2005). ERPs differentiate syllable and nonphonetic sound processing in children and adults. Psychophysiology, 42, 391–406. 16008768 10.1111/j.1469-8986.2005.00305.x

[brb3638-bib-0006] Ceponiené, R. , Torki, M. , Alku, P. , Koyama, A. , & Townsend, J. (2008). Event‐related potentials reflect spectral differences in speech and non‐speech stimuli in children and adults. Clinical Neurophysiology, 119, 1560–1577. 18456550 10.1016/j.clinph.2008.03.005 PMC2444016

[brb3638-bib-0007] Chau, W. , McIntosh, A. R. , Robinson, S. E. , Schulz, M. , & Pantev, C. (2004). Improving permutation test power for group analysis of spatially filtered MEG data. NeuroImage, 23, 983–996. 15528099 10.1016/j.neuroimage.2004.07.007

[brb3638-bib-0008] Cienkowski, K. M. , Ross, M. , & Lerman, J. (2009). The Word Intelligibility by Picture Identification (WIPI) test revisited. Journal of Educational Audiology, 15, 39–43.

[brb3638-bib-0009] Dalal, S. S. , Sekihara, K. , & Nagarajan, S. S. (2006). Modified Beamformers for Coherent Source Region Suppression. IEEE Transactions on Biomedical Engineering, 53, 1357–1363. 16830939 10.1109/TBME.2006.873752 PMC3066091

[brb3638-bib-0010] Easwar, V. , Sanfillipo, J. , Papsin, B. , & Gordon, K. (2016). Factors affecting daily cochlear implant use in children: Datalogging evidence. Journal of the American Academy of Audiology, 27, 824–838. 27885978 10.3766/jaaa.15138

[brb3638-bib-0011] Emmorey, K. , Allen, J. S. , Bruss, J. , Schenker, N. , & Damasio, H. (2003). A morphometric analysis of auditory brain regions in congenitally deaf adults. Proceedings of the National Academy of Sciences of the United States of America, 100, 10049–10054. 12904582 10.1073/pnas.1730169100 PMC187761

[brb3638-bib-0012] Fitzgerald, M. B. , Green, J. E. , Fang, Y. , & Waltzman, S. B. (2013). Factors influencing consistent device use in pediatric recipients of bilateral cochlear implants. Cochlear Implants International, 14, 257–265. 23510638 10.1179/1754762812Y.0000000026

[brb3638-bib-0013] Gilley, P. M. , Sharma, A. , & Dorman, M. F. (2008). Cortical reorganization in children with cochlear implants. Brain Research, 1239, 56–65. 18775684 10.1016/j.brainres.2008.08.026 PMC2783508

[brb3638-bib-0014] Giraud, A. L. , Price, C. J. , Graham, J. M. , & Truy, E. (2001). Cross‐modal plasticity underpins language recovery after cochlear implantation. Neuron, 30, 657–663. 11430800 10.1016/s0896-6273(01)00318-x

[brb3638-bib-0015] Gordon, K. , Abbasalipour, P. , & Papsin, B. (2016). Balancing current levels in children with bilateral cochlear implants using electrophysiological and behavioural measures. Hearing Research, 335, 193–206. 27021590 10.1016/j.heares.2016.03.013

[brb3638-bib-0016] Gordon, K. A. , Deighton, M. R. , Abbasalipour, P. , & Papsin, B. C. (2014). Perception of binaural cues develops in children who are deaf through bilateral cochlear implantation. Ed. Maurice Ptito. PLoS ONE, 9, e114841. 25531107 10.1371/journal.pone.0114841 PMC4273969

[brb3638-bib-0017] Gordon, K. A. , Henkin, Y. , & Kral, A. (2015). Asymmetric hearing during development: The aural preference syndrome and treatment options. Pediatrics, 136, 141–153. 26055845 10.1542/peds.2014-3520

[brb3638-bib-0018] Gordon, K. A. , & Papsin, B. C. (2009). Benefits of short interimplant delays in children receiving bilateral cochlear implants. Otology & Neurotology, 30, 319–331. 19318886 10.1097/MAO.0b013e31819a8f4c

[brb3638-bib-0019] Gordon, K. A. , Salloum, C. , Toor, G. S. , van Hoesel, R. , & Papsin, B. C. (2012). Binaural interactions develop in the auditory brainstem of children who are deaf: Effects of place and level of bilateral electrical stimulation. Journal of Neuroscience, 32, 4212–4223. 22442083 10.1523/JNEUROSCI.5741-11.2012 PMC6621237

[brb3638-bib-0020] Gordon, K. A. , Tanaka, S. , Wong, D. E. , Stockley, T. , Ramsden, J. D. , Brown, T. , … Papsin, B. C. (2011). Multiple effects of childhood deafness on cortical activity in children receiving bilateral cochlear implants simultaneously. Clinical Neurophysiology, 122, 823–833. 21094084 10.1016/j.clinph.2010.10.037

[brb3638-bib-0021] Gordon, K. A. , Valero, J. , & Papsin, B. C. (2007). Auditory brainstem activity in children with 9–30 months of bilateral cochlear implant use. Hearing research, 233, 97–107. 17850999 10.1016/j.heares.2007.08.001

[brb3638-bib-0022] Gordon, K. A. , Wong, D. E. , & Papsin, B. C. (2013). Bilateral input protects the cortex from unilaterally‐driven reorganization in children who are deaf. Brain, 136, 1609–1625. 23576127 10.1093/brain/awt052

[brb3638-bib-0023] Gordon, K. A. , Wong, D. E. , Valero, J. , Jewell, S. , Yoo, P. , & Papsin, B. (2011). Use it or lose it? lessons learned from the developing brains of children who are deaf and use cochlear implants to hear. Brain Topography, 24, 204–219. 21479928 10.1007/s10548-011-0181-2

[brb3638-bib-0024] Grothe, B. , Pecka, M. , & McAlpine, D. (2010). Mechanisms of sound localization in mammals. Physiological Reviews, 90, 983–1012. 20664077 10.1152/physrev.00026.2009

[brb3638-bib-0025] Hartmann, R. , Topp, G. , & Klinke, R. (1984). Discharge patterns of cat primary auditory fibers with electrical stimulation of the cochlea. Hearing Research, 13, 47–62. 6546751 10.1016/0378-5955(84)90094-7

[brb3638-bib-0026] Hine, J. , & Debener, S. (2007). Late auditory evoked potentials asymmetry revisited. Clinical Neurophysiology, 118, 1274–1285. 17462945 10.1016/j.clinph.2007.03.012

[brb3638-bib-0027] Huttenlocher, P. R. , & Dabholkar, A. S. (1997). Regional differences in synaptogenesis in human cerebral cortex. The Journal of Comparative Neurology, 387, 167–178. 9336221 10.1002/(sici)1096-9861(19971020)387:2<167::aid-cne1>3.0.co;2-z

[brb3638-bib-0028] Illg, A. , Giourgas, A. , Kral, A. , Büchner, A. , Lesinski‐Schiedat, A. , & Lenarz, T. (2013). Speech comprehension in children and adolescents after sequential bilateral cochlear implantation with long interimplant interval. Otology & Neurotology, 34, 682–689. 23640090 10.1097/MAO.0b013e31828bb75e

[brb3638-bib-0029] Jancke, L. , Wüstenberg, T. , Schulze, K. , & Heinze, H. J. (2002). Asymmetric hemodynamic responses of the human auditory cortex to monaural and binaural stimulation. Hearing Research, 170, 166–178. 12208550 10.1016/s0378-5955(02)00488-4

[brb3638-bib-0030] Jin, C. Y. , Ozaki, I. , Suzuki, Y. , Baba, M. , & Hashimoto, I. (2008). Dynamic movement of N100 m current sources in auditory evoked fields: Comparison of ipsilateral versus contralateral responses in human auditory cortex. Neuroscience Research, 60, 397–405. 18276027 10.1016/j.neures.2007.12.008

[brb3638-bib-0031] Jiwani, S. , Papsin, B. C. , & Gordon, K. A. (2016). Early unilateral cochlear implantation promotes mature cortical asymmetries in adoloscents who are deaf. Human Brain mapping, 37, 135–152. 26456629 10.1002/hbm.23019 PMC6867517

[brb3638-bib-0032] Johnson, B. W. , & Hautus, M. J. (2010). Processing of binaural spatial information in human auditory cortex: Neuromagnetic responses to interaural timing and level differences. Neuropsychologia, 48, 2610–2619. 20466010 10.1016/j.neuropsychologia.2010.05.008

[brb3638-bib-0033] Joutsiniemi, S. L. (1988). Comparison between electric evoked potentials, source dipole components and magnetic evoked fields elicited by noise/square‐wave stimuli. Acta Neurologica Scandinavica, 78, 337–345. 3223228 10.1111/j.1600-0404.1988.tb03666.x

[brb3638-bib-0034] Kaiser, J. , & Lutzenberger, W. (2001). Location changes enhance hemispheric asymmetry of magnetic fields evoked by lateralized sounds in humans. Neuroscience Letters, 314, 17–20. 11698136 10.1016/s0304-3940(01)02248-0

[brb3638-bib-0035] Kane, M. J. , & Engle, R. W. (2002). The role of prefrontal cortex in working‐memory capacity, executive attention, and general fluid intelligence: An individual‐differences perspective. Psychonomic Bulletin & Review, 9, 637–671. 12613671 10.3758/bf03196323

[brb3638-bib-0036] Key, A. P. F. , Dove, G. O. , & Maguire, M. J. (2005). Linking brainwaves to the brain: An ERP primer. Developmental Neuropsychology, 27, 183–215. 15753046 10.1207/s15326942dn2702_1

[brb3638-bib-0037] Khosla, D. , Ponton, C. W. , Eggermont, J. J. , Kwong, B. , Dort, M. , & Vasama, J.‐P. (2003). Differential ear effects of profound unilateral deafness on the adult human central auditory system. JARO, 4, 235–249. 12943375 10.1007/s10162-002-3014-x PMC3202721

[brb3638-bib-0038] King, A. J. (2009). Visual influences on auditory spatial learning. Philosophical Transactions of the Royal Society B: Biological Sciences, 364, 331–339. 10.1098/rstb.2008.0230 PMC2674475 18986967

[brb3638-bib-0039] Kotak, V. C. (2005). Hearing loss raises excitability in the auditory cortex. Journal of Neuroscience, 25, 3908–3918. 15829643 10.1523/JNEUROSCI.5169-04.2005 PMC1764814

[brb3638-bib-0040] Kotak, V. C. , Takesian, A. E. , & Sanes, D. H. (2008). Hearing loss prevents the maturation of GABAergic transmission in the auditory cortex. Cerebral Cortex, 18, 2098–2108. 18222937 10.1093/cercor/bhm233 PMC2517109

[brb3638-bib-0041] Kral, A. , Hubka, P. , Heid, S. , & Tillein, J. (2013). Single‐sided deafness leads to unilateral aural preference within an early sensitive period. Brain, 136, 180–193. 23233722 10.1093/brain/aws305

[brb3638-bib-0042] Kral, A. , & Sharma, A. (2012). Developmental neuroplasticity after cochlear implantation. Trends in Neurosciences, 35, 111–122. 22104561 10.1016/j.tins.2011.09.004 PMC3561718

[brb3638-bib-0043] Kral, A. , Tillein, J. , Heid, S. , Hartmann, R. , & Klinke, R. (2005). Postnatal cortical development in congenital auditory deprivation. Cerebral Cortex, 15, 552–562. 15319310 10.1093/cercor/bhh156

[brb3638-bib-0044] Kral, A. , Tillein, J. , Hubka, P. , Schiemann, D. , Heid, S. , Hartmann, R. , & Engel, A. K. (2009). Spatiotemporal patterns of cortical activity with bilateral cochlear implants in congenital deafness. Journal of Neuroscience, 29, 811–827. 19158306 10.1523/JNEUROSCI.2424-08.2009 PMC6665161

[brb3638-bib-0045] Lee, Y. S. , Lueders, H. , Dinner, D. S. , Lesser, R. P. , & Hahn, J. (1984). Recording of auditory evoked potentials in man using chronic subdural electrodes. Brain, 107, 115–131. 6697149 10.1093/brain/107.1.115

[brb3638-bib-0046] Litovsky, R. Y. , & Gordon, K. A. (2016). Bilateral cochlear implants in children: effects of auditory experience and deprivation on auditory perception. Hearing Research, 338, 76–87. 26828740 10.1016/j.heares.2016.01.003 PMC5647834

[brb3638-bib-0047] Magezi, D. A. , & Krumbholz, K. (2010). Evidence for opponent‐channel coding of interaural time differences in human auditory cortex. Journal of Neurophysiology, 104, 1997–2007. 20702739 10.1152/jn.00424.2009 PMC2957465

[brb3638-bib-0048] Malmierca, M. S. , & Hackett, T. A. (2010). Structural organization of the ascending auditory pathway In Rees A. & Palmer A. (Eds.), The oxford handbook of auditory science: the auditory brain, Vol. 2 (pp. 9–43). New York: Oxford University Press.

[brb3638-bib-0049] Moore, J. K. , & Guan, Y.‐L. (2001). Cytoarchitectural and axonal maturation in human auditory cortex. JARO ‐ Journal of the Association for Research in Otolaryngology, 2, 297–311. 11833605 10.1007/s101620010052 PMC3201070

[brb3638-bib-0050] O'Donnell, B. F. , Shenton, M. E. , McCarley, R. W. , Faux, S. F. , Smith, R. S. , Salisbury, D. F. , … Jolesz, F. A. (1993). The auditory N2 component in schizophrenia: Relationship to MRI temporal lobe gray matter and to other ERP abnormalities. Biological Psychiatry, 34, 26–40. 8373937 10.1016/0006-3223(93)90253-a

[brb3638-bib-0051] Pantev, C. , Ross, B. , Berg, P. , Elbert, T. , & Rockstroh, B. (1998). Study of the human auditory cortices using a whole‐head magnetometer: Left vs. right hemisphere and ipsilateral vs. contralateral stimulation. Audiology Neuro‐otology, 3, 183–190. 9575384 10.1159/000013789

[brb3638-bib-0052] Papsin, B. C. , & Gordon, K. A. (2008). Bilateral cochlear implants should be the standard for children with bilateral sensorineural deafness. Current Opinion in Otolaryngology & Head and Neck Surgery, 16, 69–74. 18197026 10.1097/MOO.0b013e3282f5e97c

[brb3638-bib-0053] Peters, B. R. , Litovsky, R. , Parkinson, A. , & Lake, J. (2007). Importance of age and postimplantation experience on speech perception measures in children with sequential bilateral cochlear implants. Otology & Neurotology, 28, 649–657. 17712290 10.1097/01.mao.0000281807.89938.60

[brb3638-bib-0054] Peters, B. R. , Wyss, J. , & Manrique, M. (2010). Worldwide trends in bilateral cochlear implantation. Laryngoscope, 120, S17–S44. 20422715 10.1002/lary.20859

[brb3638-bib-0055] Petersson, K. M. , Nichols, T. E. , Poline, J. B. , & Holmes, A. P. (1999). Statistical limitations in functional neuroimaging II. Signal detection and statistical inference. Philosophical Transactions of the Royal Society B: Biological Sciences, 354, 1261–1281. 10.1098/rstb.1999.0478 PMC1692643 10466150

[brb3638-bib-0056] Ponton, C. W. , & Eggermont, J. J. (2007). Electrophysiological measures of human auditory system maturation In Burkard R. F., Don M. & Eggermont J. J. (Eds.), Relationship with neuroanatomy and behaviour (pp. 385–402). Baltimore: Lippincott WIlliams and Wilkins.

[brb3638-bib-0057] Ponton, C. W. , Eggermont, J. J. , Khosla, D. , Kwong, B. , & Don, M. (2002). Maturation of human central auditory system activity: Separating auditory evoked potentials by dipole source modeling. Clinical Neurophysiology, 113, 407–420. 11897541 10.1016/s1388-2457(01)00733-7

[brb3638-bib-0058] Ponton, C. W. , Eggermont, J. J. , Kwong, B. , & Don, M. (2000). Maturation of human central auditory system activity: Evidence from multi‐channel evoked potentials. Clinical Neurophysiology, 111, 220–236. 10680557 10.1016/s1388-2457(99)00236-9

[brb3638-bib-0059] Quittner, A. L. , Barker, D. H. , Snell, C. , Cruz, I. , McDonald, L.‐G. , Grimley, M. E. , … Investigative Team C (2009). Improvements in visual attention in deaf infants and toddlers after cochlear implantation. Audiological Medicine, 5, 242–249.

[brb3638-bib-0060] Ramsden, J. D. , Gordon, K. A. , Aschendorff, A. , Borucki, L. , Bunne, M. , Burdo, S. , … Papsin, B. C. (2012). European Bilateral Pediatric Cochlear Implant Forum consensus statement. Otology & Neurotology, 33, 561–565. 22569146 10.1097/MAO.0b013e3182536ae2

[brb3638-bib-0061] Ross, B. (2005). Right hemispheric laterality of human 40 Hz auditory steady‐state responses. Cerebral Cortex, 15, 2029–2039. 15772375 10.1093/cercor/bhi078

[brb3638-bib-0062] Rugg, M. D. , & Coles, M. G. H. (1995). Electrophysiology of mind: event‐related brain potentials and cognition. Oxford, UK: Oxford University Press.

[brb3638-bib-0063] Salminen, N. H. , Tiitinen, H. , Miettinen, I. , Alku, P. , & May, P. J. C. (2010). Asymmetrical representation of auditory space in human cortex. Brain Research, 1306, 93–99. 19799877 10.1016/j.brainres.2009.09.095

[brb3638-bib-0064] Scheffler, K. , Bilecen, D. , Schmid, N. , Tschopp, K. , & Seelig, J. (1998). Auditory cortical responses in hearing subjects and unilateral deaf patients as detected by functional magnetic resonance imaging. Cerebral Cortex, 8, 156–163. 9542894 10.1093/cercor/8.2.156

[brb3638-bib-0065] Scherf, F. , Van Deun, L. , van Wieringen, A. , Wouters, J. , Desloovere, C. , Dhooge, I. , … Van de Heyning, P. (2009). Three‐year postimplantation auditory outcomes in children with sequential bilateral cochlear implantation. The Annals of Otology, Rhinology, and Laryngology, 118, 336–344. 10.1177/000348940911800504 19548382

[brb3638-bib-0066] Schorr, E. A. , Fox, N. A. , van Wassenhove, V. , & Knudsen, E. I. (2005). Auditory‐visual fusion in speech perception in children with cochlear implants. Proceedings of the National Academy of Sciences of the United States of America, 102, 18748–18750. 16339316 10.1073/pnas.0508862102 PMC1317952

[brb3638-bib-0067] Smith, L. B. , Quittner, A. L. , & Osberger, M. J. (1998). Audition and visual attention: The developmental trajectory in deaf and hearing populations. Developmental Psychology, 34, 840–850. 9779732 10.1037//0012-1649.34.5.840

[brb3638-bib-0068] Sparreboom, M. , Snik, A. F. M. , & Mylanus, E. A. M. (2011). Sequential bilateral cochlear implantation in children: Development of the primary auditory abilities of bilateral stimulation. Audiology and Neurotology, 16, 203–213. 20980740 10.1159/000320270

[brb3638-bib-0069] Spierer, L. , Bellmann‐Thiran, A. , Maeder, P. , Murray, M. M. , & Clarke, S. (2009). Hemispheric competence for auditory spatial representation. Brain, 132, 1953–1966. 19477962 10.1093/brain/awp127

[brb3638-bib-0070] Stecker, G. C. , McLaughlin, S. A. , & Higgins, N. C. (2015). Monaural and binaural contributions to interaural‐level‐difference sensitivity in human auditory cortex. NeuroImage, 120, 456–466. 26163805 10.1016/j.neuroimage.2015.07.007 PMC4589528

[brb3638-bib-0071] Steel, M. M. , Papsin, B. C. , & Gordon, K. A. (2015). Binaural fusion and listening effort in children who use bilateral cochlear implants: A psychoacoustic and pupillometric study. Ed. Brenton G Cooper. PLoS ONE, 10, e0117611. 25668423 10.1371/journal.pone.0117611 PMC4323344

[brb3638-bib-0072] Strelnikov, K. , Rouger, J. , Demonet, J. F. , Lagleyre, S. , Fraysse, B. , Deguine, O. , & Barone, P. (2013). Visual activity predicts auditory recovery from deafness after adult cochlear implantation. Brain, 136, 3682–3695. 24136826 10.1093/brain/awt274

[brb3638-bib-0073] Stuart, A. , Stenstromb, R. , Tompkins, C. , & Vandenhoff, S. (1991). Test‐retest variability in audiometric threshold with supraaural and insert earphones among children and adults. Audiology, 30, 82–90. 1877901 10.3109/00206099109072873

[brb3638-bib-0074] Tiihonen, J. , Hari, R. , Kaukoranta, E. , & Kajola, M. (1989). Interaural interaction in the human auditory cortex. Audiology, 28, 37–48. 2923586 10.3109/00206098909081609

[brb3638-bib-0075] Tillein, J. , Hubka, P. , & Kral, A. (2011). Sensitivity to interaural time differences with binaural implants: Is it in the brain? Cochlear Implants International, 12, 44–50. 21756472 10.1179/146701011X13001035753344

[brb3638-bib-0076] Tillein, J. , Hubka, P. , & Kral, A. (2016). Monaural congenital deafness affects aural dominance and degrades binaural processing. Cerebral Cortex, 26, 1762–1777:bhv351. 26803166 10.1093/cercor/bhv351 PMC4785956

[brb3638-bib-0077] Tillein, J. , Hubka, P. , Syed, E. , Hartmann, R. , Engel, A. K. , & Kral, A. (2010). Cortical Representation of Interaural Time Difference in Congenital Deafness. Cerebral Cortex, 20, 492–506. 19906808 10.1093/cercor/bhp222

[brb3638-bib-0078] Vrba, J. , & Robinson, S. E. (2001). Signal Processing in Magnetoencephalography. Methods, 25, 249–271. 11812209 10.1006/meth.2001.1238

[brb3638-bib-0079] Weeks, R. , Horwitz, B. , Aziz‐Sultan, A. , Tian, B. , Wessinger, C. M. , Cohen, L. G. , … Rauschecker, J. P. (2000). A positron emission tomographic study of auditory localization in the congenitally blind. Journal of Neuroscience, 20, 2664–2672. 10729347 10.1523/JNEUROSCI.20-07-02664.2000 PMC6772250

[brb3638-bib-0080] Wilke, M. , Holland, S. K. , Altaye, M. , & Gaser, C. (2008). Template‐O‐Matic: A toolbox for creating customized pediatric templates. NeuroImage, 41, 903–913. 18424084 10.1016/j.neuroimage.2008.02.056

[brb3638-bib-0081] Wong, D. E. , & Gordon, K. A. (2009). Beamformer suppression of cochlear implant artifacts in an electroencephalography dataset. IEEE Transactions on Biomedical Engineering, 56, 2851–2857. 19695980 10.1109/TBME.2009.2029239

[brb3638-bib-0082] Yamazaki, H. , Easwar, V. , Polonenko, P. , Jiwani, S. , Wong, D. E. , Papsin, B. , & Gordon, K. (unpublished data) Development of right hemispheric specialization to monaural tone‐bursts from early childhood.

[brb3638-bib-0083] Yucel, E. , & Derim, D. (2008). The effect of implantation age on visual attention skills. International Journal of Pediatric Otorhinolaryngology, 72, 869–877. 18395272 10.1016/j.ijporl.2008.02.017

[brb3638-bib-0084] Zatorre, R. , & Belin, P. (2001). Spectral and temporal processing in human auditory cortex. Cerebral Cortex, 11, 946–953. 11549617 10.1093/cercor/11.10.946

[brb3638-bib-0085] Zeitler, D. M. , Kessler, M. A. , Terushkin, V. , Roland, T. J. , Svirsky, M. A. , Lalwani, A. K. , & Waltzman, S. B. (2008). Speech perception benefits of sequential bilateral cochlear implantation in children and adults: A retrospective analysis. Otology & Neurotology, 29, 314–325. 18494140 10.1097/mao.0b013e3181662cb5

[brb3638-bib-0086] Zheng, Y. , Godar, S. P. , & Litovsky, R. Y. (2015). Development of sound localization strategies in children with bilateral cochlear implants. Ed. Frederic Dick. PLoS ONE, 10, e0135790. 26288142 10.1371/journal.pone.0135790 PMC4545829

[brb3638-bib-0087] Zwiers, M. P. , Van Opstal, A. J. , & Cruysberg, J. R. (2001). A spatial hearing deficit in early‐blind humans. Journal of Neuroscience, 21, 1–5. 10.1523/JNEUROSCI.21-09-j0002.2001 PMC6762556 11312316

